# Direct observation of single organic molecules grafted on the surface of a silicon nanowire

**DOI:** 10.1038/s41598-019-42073-5

**Published:** 2019-04-04

**Authors:** Rosaria A. Puglisi, Sebastiano Caccamo, Corrado Bongiorno, Giuseppe Fisicaro, Luigi Genovese, Stefan Goedecker, Giovanni Mannino, Antonino La Magna

**Affiliations:** 1 Istituto per la Microelettronica e Microsistemi, Consiglio Nazionale delle Ricerche, Catania, 95121 Italy; 2 Laboratoire de simulation atomistique (L_Sim), SP2M, INAC, CEA-UJF, F-38054 Grenoble, France; 3 Department of Physics, University of Basel, Klingelbergstrasse 82, CH-4056 Basel, Switzerland

## Abstract

Silicon nanowires inspire since decades a great interest for their fundamental scientific importance and their potential in new technologies. When decorated with organic molecules they form hybrid composites with applications in various fields, from sensors to life science. Specifically the diethyl 1-propylphosphonate/Si combination is considered as a promising alternative to the conventional semiconductor n-type doping methods, thanks to its solution-based processing, which is damage-free and intrinsically conformal. For these characteristics, it is a valid doping process for patterned materials and nanostructures such as the nanowires. Our joined experimental and theoretical study provides insights at atomistic level on the molecular activation, grafting and self-assembling mechanisms during the deposition process. For the first time to the best of our knowledge, by using scanning transmission electron microscopy the direct visualization of the single molecules arranged over the Si nanowire surface is reported. The results demonstrate that the molecules undergo to a sequential decomposition and self-assembling mechanism, finally forming a chemical bond with the silicon atoms. The ability to prepare well-defined molecule decorated Si nanowires opens up new opportunities for fundamental studies and nanodevice applications in diverse fields like physics, chemistry, engineering and life sciences.

## Introduction

Several decades after their introduction Si nanowires (SiNWs) still represent the subject of a vast literature, reaching a publication record of more than 1500 papers per year (http://wcs.webofknowledge.com). Such an extended interest in the material itself, is justified by its extraordinary optical and electronic properties as well as its potential technological applications ranging from nanoelectronics to photovoltaics for the industrial sector^1–6^. When the SiNWs surface is functionalized with organic molecules, it forms a hybrid nanosystem exhibiting other properties and functionalities such as surface passivation and tunable wettability^7^. This hybrid structure finds exciting application in life sciences, where it has been proposed as next-generation therapeutic devices, as analytical tool to decipher how neurons store and process information, or for recording intracellular bioelectrical signals to understand the cells and cell-networks behavior in neural and cardiac systems^8–14^. Thanks to their large surface-to-volume ratio and three-dimensional multi-gate structure, SiNWs find also utilization in the sensors area, for the high sensitivity they offer compared to conventional planar devices, and for the potentiality of label-free detection of chemical and biological species^15^.

The chemical properties of the bare or functionalized SiNW surface play a strategic role depending on the species to be sensed. The presence of an SiO_x_ layer at the surface of the SiNW is in literature exploited for the detection of protons and gases^16^. For the biomolecules detection, an affinity carbon-based layer, placed on top of the Si pristine NW surface and interacting with the analyte of interest, is generally proposed^15^.

The organic molecule diethyl 1-propylphosphonate (DPP) is used as dopant vector and presents the additional property to release, upon high temperature annealing, phosphorous atoms which subsequently diffuse from the surface towards the Si bulk, where they work as dopants^17^. This makes the system DPP/Si a unique combination to be exploited in the semiconductor field as an alternative to the standard doping methods. The technique taking advantage of these properties is known in literature as Molecular Doping or Monolayer Doping (MD) and has found a growing interest in the semiconductor community for its easiness, low cost and efficacy^18–32^. Since it is based on solution processing, the method is intrinsically suitable for conformal doping, i.e. a process where the dopant atoms follow the surface of 3-dimensional nanostructures or hollow structures.

The mechanisms underlying the molecule positioning on the Si surface have been preliminarily studied in literature. It is known that in MD, when the allylboronic acid pinacol ester is used, it binds to the surface of the planar Si, forming a covalent bond through the carbon atom^17^. Concerning the DPP molecule, studies based on surface chemical analysis preliminarily suggest that the molecule decomposes during deposition and sticks to Si planar surface through oxygen atoms^20^. However, these studies are based on standard chemical analysis, which gives average information on a large portion of the surface. There is still no detailed and in-depth information on how the molecule is bound and on its high-resolution visualization.

The interaction between the molecule and the substrate is the key aspect to be understood to control the final chemical, physical and electrical characteristics of the hybrid nano-systems. For this reason, the prerequisite for a real progress in any type of application, is then an in-depth understanding of what happens at atomic scale when the molecule arrives on the Si surface and how it arranges on it. The direct observation of the single molecules is however broadly recognized in the scientific world as an arduous challenge because of different reasons: the difficulty to isolate the single object rather than an ensemble; the limited resolution of the available characterization techniques or the molecule possible modification when prepared for high resolution microscopy analysis; above all the difficulty to couple the structural and morphological information at nm level to the chemical composition data.

Through a high resolution direct observation, our work presents for the first time results on a structural and chemical investigation on single DPP molecules right after their grafting on the SiNW surface. The Si nanostructures have been synthesized by plasma-assisted chemical vapour deposition (CVD) catalyzed by gold nanoclusters^6^. The organic molecule has been deposited over the SiNW surface where it self-assembled in a single layer. This enabled the isolation of single molecules along the observation direction, without superposition of molecular multi-layers. The type of instrument used permitted to acquire Transmission Electron Microscopy (TEM) dark field images through a High Angle Annular Dark Field (HAADF) detector, allowing to simultaneously perform electron energy loss and energy dispersive X ray spectroscopies. The data have all been collected in a single multi-dimensional data matrix with sub-nanometer precision, to provide structural, morphological and chemical information at high resolution.

Details on the molecule decomposition and grafting mechanisms lying at the basis of the DPP/Si bonding process, have been obtained at atomic level by means of density functional theory (DFT) calculations. We investigate the interaction between the molecule and the SiNW applying ab-initio structural searches on the full configurational space. The coupling between the simulations and the highly resolved chemical and structural results provides a clear visualization of the molecular layer and an in depth understanding of the atomic arrangements at the interface of this hybrid system.

## Results and Discussion

**Figure 1:**
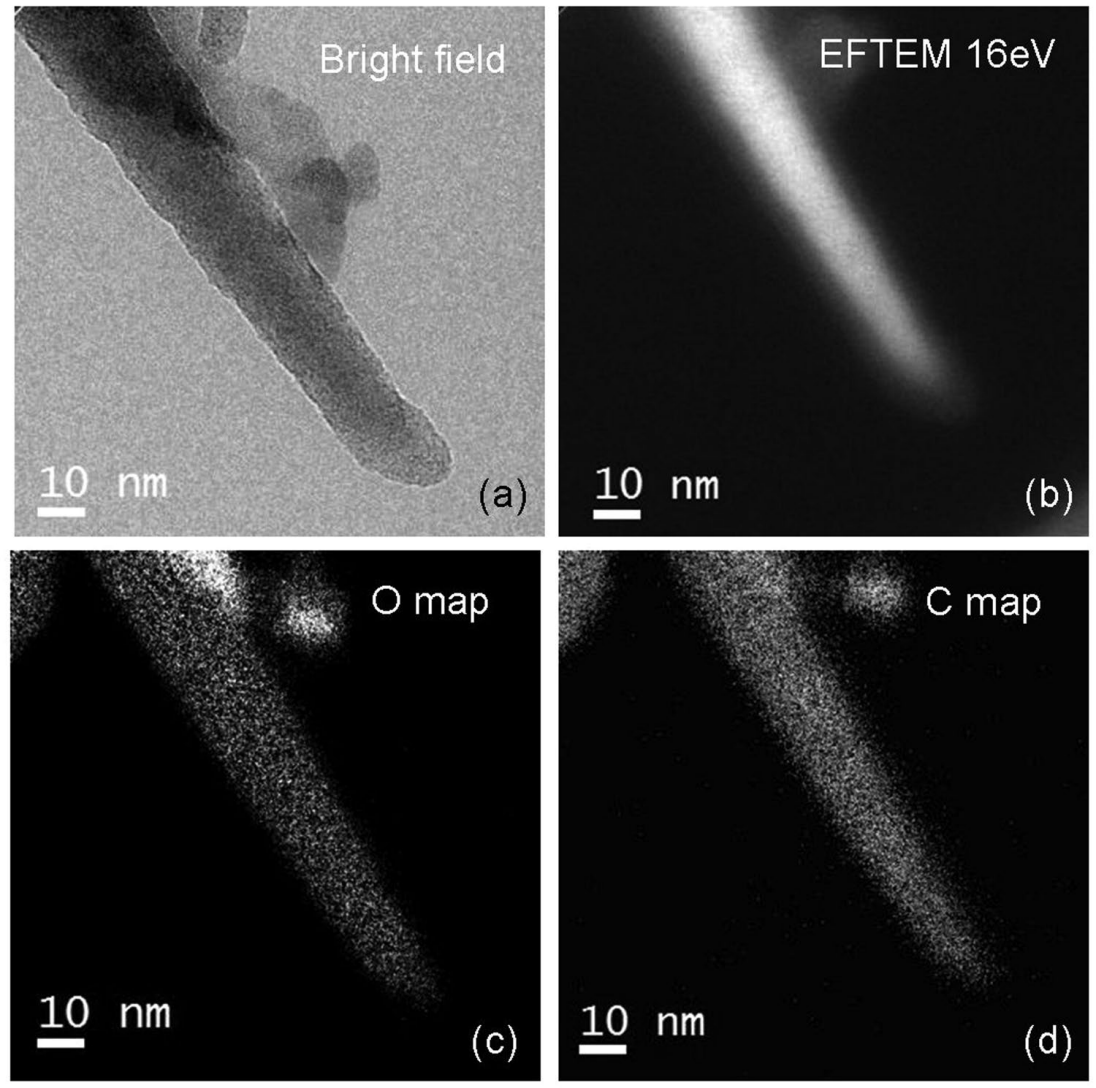
Structural and chemical characterization of the Si nanowires. (**a**) Bright field TEM micrograph of a NW after the molecules deposition; (**b**) EFTEM image taken at 16 eV; (**c,d**) O and C maps acquired on the same nanostructure”.

After the deposition of the DPP molecules, the SiNWs were collected on a TEM grid and analyzed. Figure 1(a) reports the image of a SiNW after the molecules attachment, acquired through bright field TEM analysis. Under this conditions the image contrast observed in the micrograph is independent on the chemical phases present inside and on the surface of the SiNW. In order to distinguish between the chemical species, the *in situ* Energy Filtered TEM (EFTEM) analysis has then been applied. Figure 1(b) illustrates the EFTEM micrograph collected in the EELS mode. The energy-selecting window used to image the SiNW was 4 eV centered at 16 eV energy loss, i.e., close to the Si bulk plasmon value. Due to the difference in plasmon energy loss between Si and SiO_2_, during the acquisition only the bulk Si regions are imaged^33^. As it is evident from the comparison with Fig. 1(a), in this micrograph the SiNW diameter appears smaller than in the corresponding bright field image. This suggests that the SiNW presents a Si core and a shell with a chemical composition to be further investigated. Figure 1(c,d) reports the O and C maps acquired on the same region. From the filtered images it is evident that the C and O signals are broader than the Si one, thus suggesting the presence of layers made of these materials, surrounding the SiNW.

As it is evident, the low signal-to-noise ratio of this type of analysis makes impossible to distinguish with sub-nm accuracy the position of the different species on the NW walls, neither to visualize the presence of the deposited molecules over the nanostructure surface.

**Figure 2:**
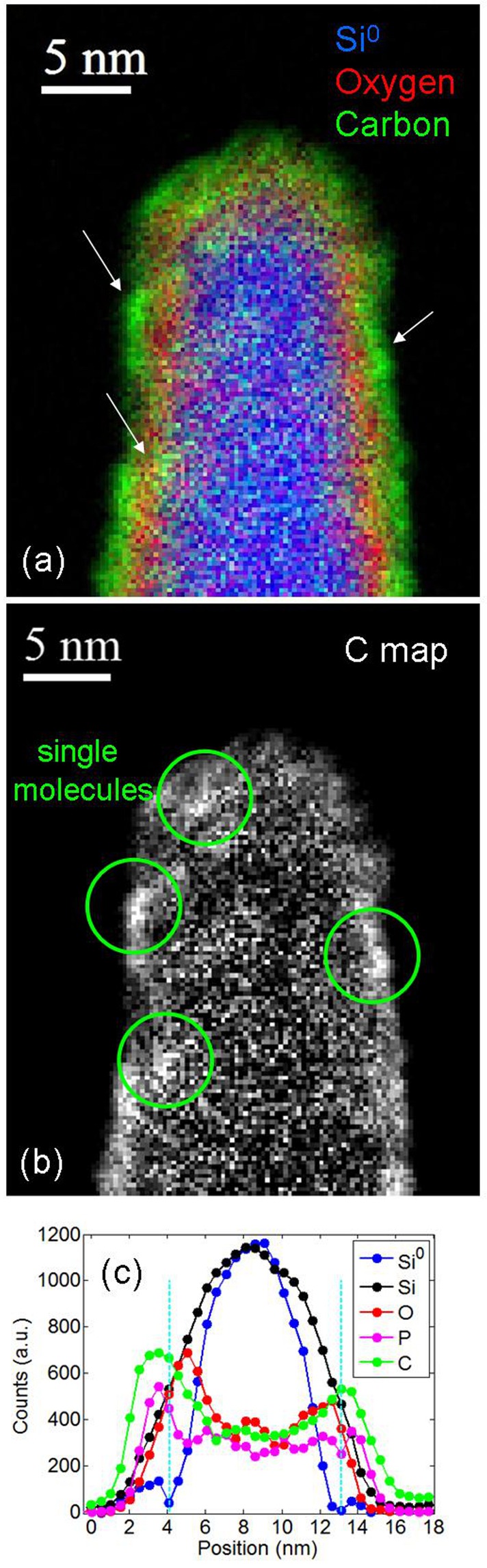
Spectrum imaging in STEM mode of Si nanowires decorated by ester molecules. (**a**) RGB image showing the region of a NW close to the tip, composed by the superposition of the Si, O and C two-dimensional maps (blu, red and green respectively) extracted from the EELS spectrum image data-cube. (**b**) C map where the single molecules on the NW surface are indicated by the green circles. (**c**) Chemical profiles extracted by the spectrum image and by the EDX signal, referring to the distribution profiles of Si-Si bonds (blue data), total Si (black), oxygen (red), carbon (green) and phosphorous (magenta). The two dashed lines indicate the position of the C-O interface”.

For this reason the nanostructures have been observed at high magnification by using the spectrum imaging Scanning TEM (STEM) mode. The final results of this investigation are illustrated in Fig. 2(a). It shows the region of a NW close to its top. The red-green-blue (RGB) image represents the superposition of the Si, O and C two-dimensional maps (blue, red and green respectively) extracted from the same EELS spectrum image data-cube. The source microscopies can be found in the Supplementary File, Fig. S1. It is worth to note that with respect to the maps obtained by EFTEM imaging, the two-dimensional maps acquired through the STEM technique, thanks to the small size and high intensity of the e-beam, and consequent high signal-to-noise ratio, allow for high spatial resolution and self-aligned elemental 2D maps acquisition.

**Figure 3:**
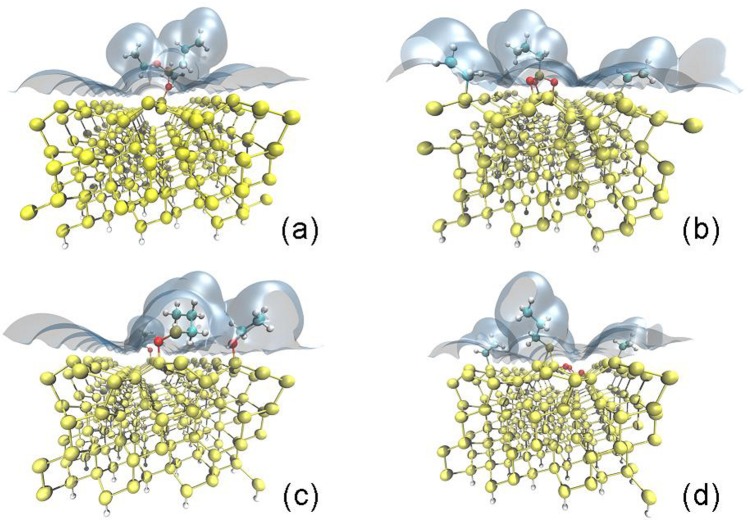
Theoretical DFT results on the DPP/Si interaction. (**a**) DPP original un-broken molecule on the Si surface. (**b**) DPP molecule interacting with the Si surface and dissociating the -CH_2_-CH_3_ groups (case I); (**c**) with dissociated -O-CH_2_-CH_3_ (case II). (**d**) Global DFTB+ minima hopping minimum where the DPP molecule is dissociated and the detached -CH_2_-CH_3_ groups and O atoms move away. Yellow, red, grey, blue and white spheres represent, respectively, Si, O, P, C and H atoms. The regions in light blue indicate an iso-surface of the dielectric function ɛ(r) = 2, indicating the transition region between the quantum system and the continuum embeddings modelling the mesitylene”.

Due to the 3-dimensional geometry of the analyzed nanostructure and the consequent integration effect on the side walls of the SiNW, the signal increases in correspondence of the lateral curvature. The resulting image clearly evidences the separation between the two layers of O and C, red and green respectively in the image, revealing that they form two different shells. The oxygen layer is placed between Si and C and appears rough but continuous with a thickness of about 1.2 nm. This thickness can be overestimated due to the 3-dimensionality of the structure and to the consequent curvature effect on the lateral walls of the NW. Its presence may be attributed to the native oxide regrowth after the SiNW air exposure, before the DPP deposition despite the immediacy of the sample transfer, and/or to a contribution from the molecular oxygen atoms. This last result could preliminary confirms the previous investigations performed on planar Si surfaces functionalized by DPP^20^, suggesting that the molecule is bound via the oxygen to the Si substrate^20,34^. To further deepen this aspect, the analysis was focalized also on the other atoms’ signal and the results will be discussed below also in combination with the simulation outcomes (see Fig. 3(d) and discussion in the following).

The C signal illustrated by the green color in Fig. 2(a) is clearly not continuous and exhibits the presence of agglomerates with nm size (white arrows in the figure). This result is more evident in Fig. 2(b) reporting the C map of the same NW. The C agglomerates are evidenced by the green circles. To understand if the observed features can be ascribed to the presence of the single molecules, the molecular footprint has been estimated by molecular modeling by using the Avogadro software tool and the results are reported in the Supporting Information, Fig. S3. The molecule footprint calculated with this method results to be 0.24 nm^2^.

Since during the self-assembling deposition process the molecules experience the Van der Waals (VdW) forces, the calculation of their effective footprint has to take them into account. The corresponding maximum lateral sizes and area, considering the VdW surfaces, result then to be respectively: 1.3 nm, 1 nm and 0.49 nm^2^. These values are dimensionally compatible with the size of the single molecules observed in Fig. 2(b).

Figure 2(c) illustrates the chemical profiles extracted by the spectrum image, averaging over 15 nm in a region far from NW tip curvature. The blue and black data refer respectively to the Si-Si bond and to the total Si distribution profiles. The difference in width between the two curves confirms the presence of the SiO_x_ shell around the Si core. The red and green curves report the oxygen and carbon profiles and show the spatial separation between the two shells above mentioned. The two dashed lines indicate the position of the C-O interface.

Regarding the phosphorous signal, since there is only one P atom per molecule the signal is relatively low to be revealed in a 2D map, so the P profile has been extracted by the EDX signal, whose source signal is reported in the Supplementary File, Fig. S2. The average line profile allowed to integrate over a larger number of molecules deposited over the NW surface. The magenta curve in Fig. 2(c) shows the integration profiling results. They demonstrate that the P dopant atoms are placed in correspondence of the C-O interface. As in the case of the RGB imaging, the integration effect plays a role also in the data profiling. The O, P and C signals detected between 5 and 11 nm of the x axis, i.e. in correspondence of the NW core center, are due to the atoms present on the top and back surface of the NW walls facing the e-beam. So they are due the 3-dimensional shape of the observed nanostructure and to the consequent integration effect on these atoms. This result is hence not a demonstration that the O, P and C atoms are present inside the NW core. Previous studies have been conducted to investigate the composition of SiNWs, by the 2-dimensional EELS spectra^35^. In that case, the presence of the atoms was revealed by the spectra, and not directly visualized through imaging like in the present work. Here we are demonstrating the direct visualization of the organic molecules imaged at high resolution by selecting and identifying only the carbon species, as well as for the P and O, through a technique associating the 2-dimensional analysis to the third dimension, i.e. the chemical information, acquired in the precise position investigated.

In order to obtain details on the atomic mechanism at the basis of the functionalization, a theoretical DFT study^36,37^ of the interaction between the DPP molecule and the (111) Si surface has been carried out and the results reported in Fig. 3(a–d). Yellow, red, grey, blue and white spheres represent, respectively, Si, O, P, C and H atoms.

DFT simulations takes into account the presence of the mesitylene solvent by means of the soft-sphere model^38,39^. The regions in light blue indicate an iso-surface of the dielectric function ɛ(r) = 2, indicating the transition region between the quantum system and the continuum embeddings modelling the mesitylene.

To get insights into the interaction of the molecule and the Si wet surface, several configurations molecule/surface have been optimized at DFT level. In the first set of calculations the molecule is not broken at any bonds with respect to its isolated conditions. Input geometries have been carried out rotating the molecule in such a way that only one, two or all three oxygens of the unbroken molecule were initially located in proximity of surface Si atoms. During the DFT geometry optimizations, the molecule turns and moves in order to maintain only a single bond between the free oxygen of the molecule (the one not bound to any -CH_2_-CH_3_ chains) and a Si surface atom. To quantify the interaction between the molecule and the substrate, the molecule binding energy Δ*E*_*B*_, defined as the difference of the total energy of the system (molecule + surface) and their energies in isolated conditions, has been computed both in vacuum and mesitylene. All energetics have been extracted after relaxation. The energetics order has been preserved between vacuum and solvent calculations.

Figure 3(a) reports the lowest energy configuration of the molecule in the unbroken state and interacting with the Si surface in mesitylene. One oxygen is bound to a Si atom belonging to an internal arrow, partially displacing it from its initial site. The binding energy of this configuration is positive (Δ*E*_*B*_ = 197 meV, a value even larger than the one obtained in vacuum Δ*E*_*B*_ = 63 meV). This simulation result suggests that the binding process of an unbroken molecule to the surface is infeasible and the solvent presence actually makes it even more difficult.

A dissociative reaction pathway should then occur and two possible pathways have been explored: (case I) the breaking of -CH_2_-CH_3_ chains, allowing for three oxygen bonds with three distinct Si atoms; (case II) the breaking of the two -O-CH_2_-CH_3_ chains, leaving the possibility of a single O-Si bond between the free oxygen of the molecule and a Si atom or/and a P-Si bonding. The dissociated parts of the molecule have been then placed in proximity of the surface in the DFT optimization study. Figure 3(b) reports the local DFT minimum configuration for the case (I) detaining a binding energy of Δ*E*_*B*_ = −3.04 eV. Such huge binding energy supports the idea that molecule breaks once it meets the Si surface and that the breaking occurs at the O-C level, leaving to the three oxygens the possibility to covalently bond to the surface.

The binding energy of the local DFT minimum configuration for case (II), showed in Fig. 3(c), is Δ*E*_*B*_ = −0.31 eV. This negative binding energy suggests their feasibility, although is energetically unfavorable with respect to case I.

To fully explore the configuration space, we employed the minima hopping method^40,41^ coupled to DFTB+^42^ to get energy and forces at an affordable computational cost. The DFTB+ search of the global minimum confirms the high stability of configurations which detain a bond break at the O-C level, leaving three oxygens covalently bond to the surface (for the details see Supporting Information). The global minimum structure is reported in Fig. 3(d). In the solvent this configuration is quasi-degenerate with the respect to the one shown in Fig. 3(b) (with a very small activation energy of 0.04 eV). It is noteworthy for the MD mechanism that, in this configuration, the three O atoms are instead embedded in the surface matrix (each one between two silicon atoms) and the P atom is covalently bonded to two surface sites. This configuration could represent one of the steps involved in the phosphorous migration during MD process. Minima hopping also found all intermediate states where the phosphorus atom is bound to two or one oxygen, suggesting that first the molecule dissociate from -CH_2_-CH_3_ groups, and then adsorbs to the surface with three O-Si bonds. Afterwards, the phosphorous atom leaves all oxygens one by one and bonds to two surface site.

The simulations results are coherent with the Raman analysis of the Si after the molecular doping treatment, revealing the presence of propionate groups. Such experimental evidence suggested a break of the molecule once it meets the Si surface and the rearrangement of the O atoms at the interface with the Si^20^. The whole picture provided by the experiments and the simulations yet confirms the STEM profiles of Fig. 2(c) showing unequivocally the O position between the P and the Si signals.

It is worth to note that if the molecule binds to Si through O and not through C atoms, a uniform and compact self-assembly can be expected. If instead it would have bound to Si through C, the strong C-Si bond would have delayed the surface diffusion of the molecules once arrived on the surface, and consequently the complete coverage and formation of the compact monolayer disadvantaged. Thanks to this decomposition and rearrangement mechanism the molecules density and relative distance are determined by their steric properties and can be controlled in a deterministic way. The discovery of how many molecules can be grafted and how they attach themselves has a direct impact on the control of the NW doping and as a consequence on how to modulate its conductivity, once they are diffused inside the nanostructure. This will make the doped nanowires an ideal platform for a wide variety of applications. Furthermore, the same knowledge of how the molecules bind to the hybrid interface, makes the nano-system extremely attractive as a biosensor with biomolecular recognition properties, chemical stability, and interfacial electrical properties. Finally it will be possible to improve the SiNW properties such as surface passivation and wettability.

## Conclusions

One layer of the organic ester diethyl 1-propylphosphonate molecule has been deposited by solution processing on the SiNWs surfaces and observed by STEM. Single molecules have been for the first time observed. The high resolution chemical and structural investigation has let understand that the SiNW core is covered by an oxygen shell, a P interlayer and a carbon external layer. This external layer evidences agglomerates to be ascribed to the single DPP molecules. The phosphorous signal is found in correspondence with the O-C interface. This clearly indicates that the molecule undergoes to a modification of its structure upon grafting to the SiNW. The bonding mechanism of DPP to the Si surface has been also theoretically studied by ab-initio structure predictions, taking into account the solvent ambient, and the results suggest that the molecule interacts with the substrate and dissociates once it meets Si surface. The breaking occurs at the oxygen-carbon level, leaving the three residual oxygen atoms covalently bonded to the Si substrate, thus confirming the experimental observations.

## Methods

### Experimental Method

For the synthesis of SiNWs, 6″ <100> p type 1–5 ohm*cm Si wafers were HF etched and introduced into a K675XD magnetron sputter to deposit 1.1 × 10^16^ at/cm^2^ of gold with 15 mA for 20 s at 5 × 10^−3^ mbar, with ~10^11^ cm^−2^ final Au dot density. After a further HF dip the wafer was immediately loaded into a Inductively Coupled Plasma Chemical Vapor Deposition (ICPCVD). The deposition took place for 30 min at 395 °C, 20 W, 20 mTorr, by using a gas ratio SiH_4_/Ar = 30. The wafer was then subjected to a gold etch process performed with HF etch, water rinse, immersion in a solution of Fujifilm gold etch II w/OHS, Sodium iodide NAI and Iodine, rinsed again and blow dry. The MD process started with a HF dip of the SiNWs samples, cut into pieces of 1 × 1 cm^2^, to remove native oxide, followed by immersion in a solution of diethyl 1-propylphosphonate and mesitylene (20% v/v) at about 160 °C for 2.5 h. Then the samples were analyzed by TEM in standard and scanning modes. EFTEM analysis were obtained with a JEOL JEM 2010F machine operating at 200 kV equipped with a Schottky field emission gun (FEG) and a post-column Gatan Imaging Filter. To acquire the images, an electron beam acceleration energy of 60 keV has been used. The dose level was chosen in order to acquire a signal for the chemical analysis sufficient to visualize the position of the molecules. The energy filtering system is a Gatan GIF based on a magnetic-prism spectrometer and a 2 k × 2 k multiscan CCD camera.

### Simulation Method

The simulations have been performed by means of first-principle electronic-structure calculations. Kohn-Sham density functional theory has been employed within the BigDFT package^36,37^. BigDFT allows treating exactly free, surface and periodic boundary conditions as well as the inclusion of complex wet environments by means of the soft-sphere continuum solvation model^38,39^. The relative dimensions of the molecule with respect to the SiNW allowed us to modelled the molecule-SiNW system as a single molecule on top of a Si substrates. A (111) surface with a 2 × 1 reconstruction has been considered.

We explored the configurational space of the molecule-surface system by means of the minima hopping (MH) method^40,41^. To speed up the structural search, we coupled MH to density functional tight binding (DFTB+)^42^. DFTB+ guarantees a faster computation of energies and forces with respect to *ab-initio* DFT, maintaining a reasonable accuracy. MH provides the exploration of the potential energy surface of a target atomistic system, finding minimum energy structures by means of molecular dynamics trajectories and without restrictions on molecular bond breaking. Subsequently, several DFTB+ configurations have been optimized at DFT level. To quantify the interaction between the molecule and the substrate, we computed the molecule binding energy Δ*E*_*B*_ both in vacuum and mesitylene, defined as the difference between the total energy of the system (molecule + surface) and their energies in isolated conditions. Technical details of the DFT calculations are reported in the Supporting Information.

## Supplementary information

Supplementary File
