# Supplementary material for: Direct observation of single organic molecules grafted on the surface of a silicon nanowire
Source: Sci Rep. 2019 Apr 4;9:5647. doi: 10.1038/s41598-019-42073-5 (PMC6449362; doi:10.1038/s41598-019-42073-5)
Supplement: Supplementary file 1 — Supplementary File [file 41598_2019_42073_MOESM1_ESM.doc]

**SUPPORTING INFORMATION**

**Direct observation of single organic molecules grafted on the surface of a silicon nanowire**

Rosaria A. Puglisi*, Sebastiano Caccamo, Corrado Bongiorno, Giuseppe Fisicaro, Luigi Genovese, Stefan Goedecker, Giovanni Mannino, and Antonino La Magna

* E-mail: rosaria.puglisi@imm.cnr.it

The energy filtered images at 16 eV were acquired by selecting in the electron energy loss spectrum (EELS) the energy window characteristic of bulk Si plasmon loss. Chemical maps for Si, C and O elements were acquired at 100, 285 and 535 eV respectively, using the three windows method for the background subtraction. The final image provides an intensity map correlated to the amount of the monitored chemical element. Spectrum imaging in the scanning mode (STEM) was taken at 60kV with JEOL ARM 200, equipped with a cold FEG, aberration corrected condenser lens, JEOL 100 mm^2^ Energy Dispersive X-ray (EDX) detector and Gatan Quantum Spectrometer. The instrument provides during a single scan a dark field image with High Angle Annular Dark Field (HAADF) detector, simultaneously with EELS and EDX spectra, all collected in a single multi-dimensional data matrix. The RGB image consists in the superposition of 3 chemical maps of Si, O and C, obtained from the same EELS spectrum image data-cube. The energy windows used to acquire the Si map were in the range of 99 – 104 eV, corresponding to the not oxidized Si-Si bonds, and in the range 99 – 125 eV, comprehensive of the oxidized species. In order to reduce the signal/noise ratio, the elemental profiles were calculated by integrating the signal on a 15 nm wide area far from the tip. This methodology allowed to extract also the low intensity EDX phosphorous signal. To avoid chemical modifications during standard specimen thinning procedures, the NWs were mechanically collected on a carbon coated copper grid, right after the doping step.


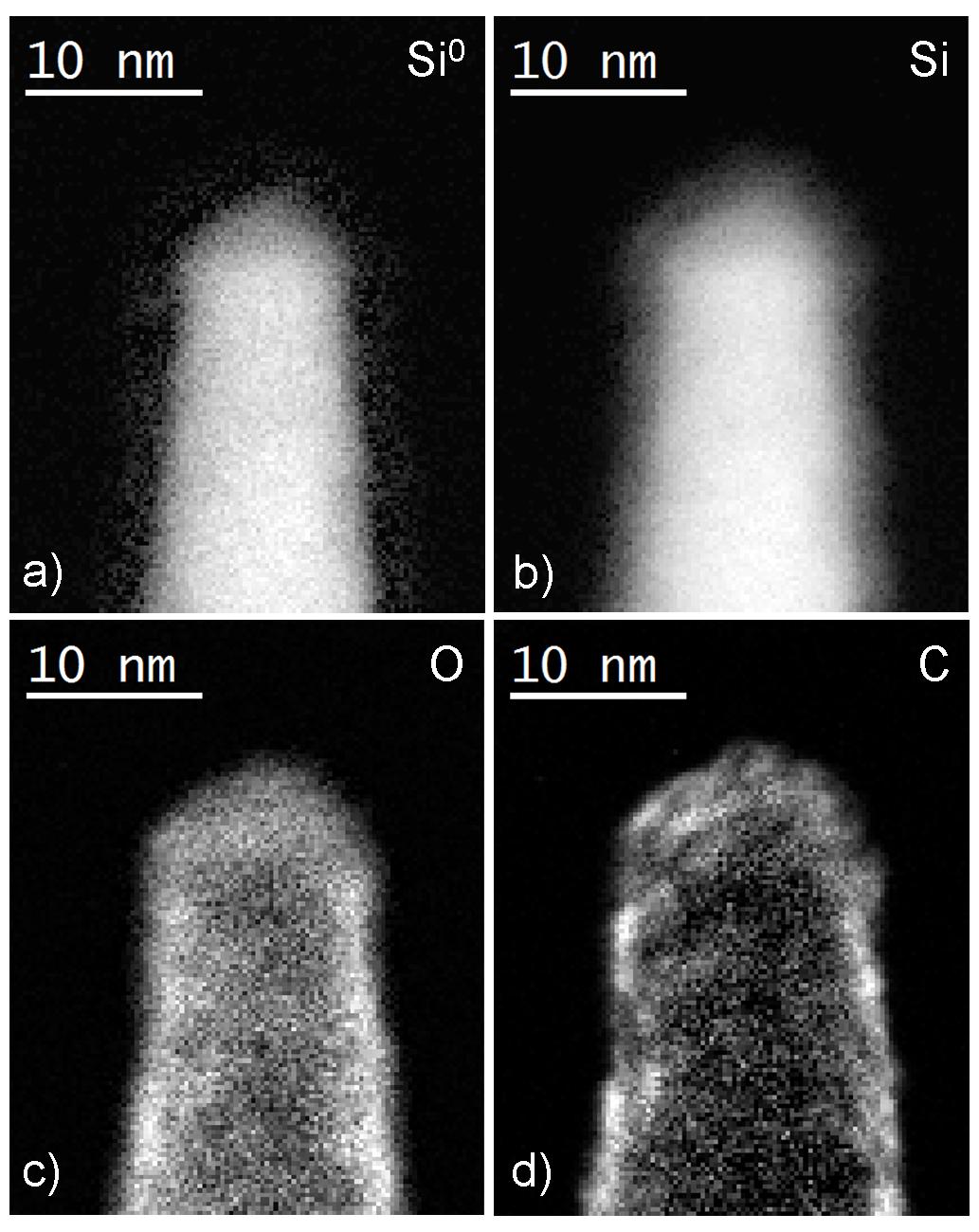


**Supporting Figure 1 |** **Single spectrum imaging chemical maps.** Maps relative to the Si^0^ (**a**), Si (**b**), O (**c**) and C (**d**). The Si^0^ (**a**), O (**c)** and C (**d**) represent the source images of the RGB composition reported in Figure 2 **a** of the manuscript.


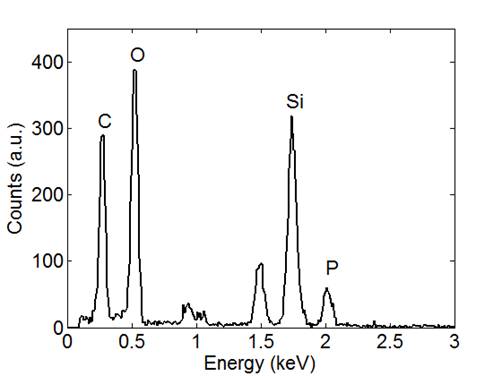


**Supporting Figure 2 |**. **EDX spectrum.**  Spectrum obtained by integration on a 20 nm × 5 nm NW area, corresponding to the nanostructure sidewall. The P peak, at 2.1 keV, is the source signal of the P profile shown in Figure 2 **c** of the manuscript. The C, O and Si peaks are visible at 0.3, 0.5 and 1.7 keV. The peaks at 0.9 and 1.5 keV correspond respectively to Cu and Al due to the sample and detector holders.


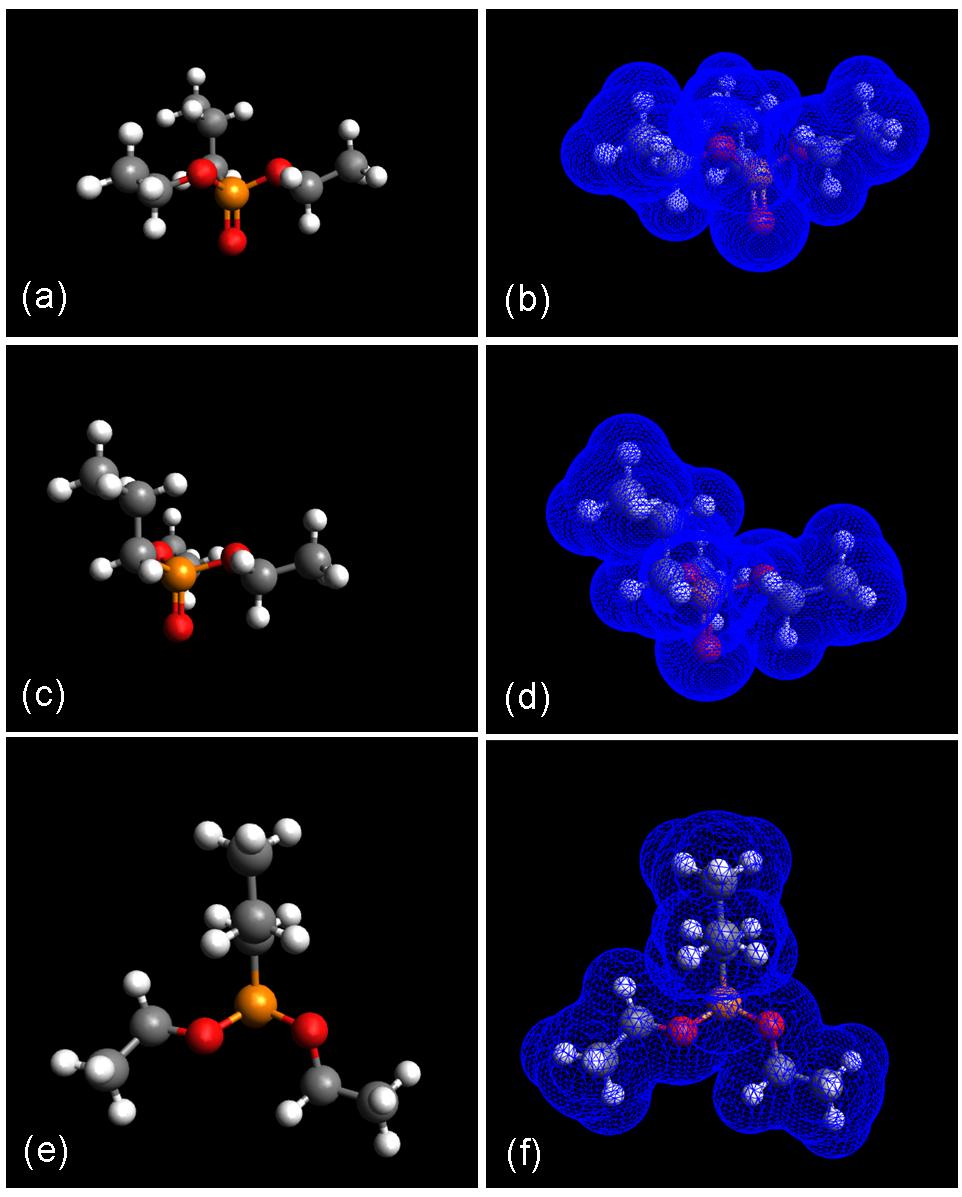


**Supporting Figure 3 |** **Results of the Avogadro modeling.** Modeling of the pristine DPP molecule structure (**a**, **c**, **e**) with corresponding Van der Waals surfaces (**b**, **d**, **f**) obtained by using Avogadro software based on the universal force field approximation. From these structures the molecule maximum lateral extension **a**, and the minimum lateral extension **c**, have been calculated and are respectively 0.9 nm and 0.7 nm. **e** The molecule footprint approximating its projection shape to a triangle is 0.24 nm^2^. The corresponding maximum lateral sizes and areas taking into account also the Van der Waals surfaces result to be: 1.3 nm (**b**), 1 nm (**d**) and 0.49 nm^2^.

**SUPPORTING TEXT**

**First-principle calculations**

Regarding the first-principle electronic-structure calculations, Kohn-Sham density functional theory (DFT) has been employed within the BigDFT package^1,2^. BigDFT allows to treat exactly free, surface and periodic boundary conditions as well as the inclusion of complex wet environments in an implicit way. DFT computations are done at 0 K. Although they do not include the entropy contribution to the surface energy, results still represent a good reference to assess relative stabilities. Soft norm-conserving pseudopotentials including non-linear core correction^3,4^ along with PBE functional were used to describe the core electrons and exchange-correlation for all calculations. To make energetic comparisons, we performed all runs with equivalent parameter setting and convergence criteria. All structures have been relaxed using the Hellman-Feynman scheme until all the forces are less than 0.01 eV/Ȧ.

A Si <111> surface has been considered with a 2 × 1 reconstruction to take into account the crystallographic orientation of the planar Si substrates used in the experiment. The structure has been built from its orthorhombic supercell with initial sizes of 3.86 × 6.69 Ȧ in the orthogonal directions *x* and *y* of the surface plane (*z* is orthogonal to the surface). To avoid spurious interactions with periodic images of the organic molecule in the periodic directions *x* and *y*, the cell has been extended by 5 and 3 times, respectively, getting a final surface of 19.33 × 20.09 Ȧ (total 270 Si atoms). Initially a four layers slab has been optimized in vacuum at density functional theory level. Surface boundary conditions have been set for all surface calculations and last 2 layers have been frozen to reproduce bulk conditions. One surface is considered in contact with mesitylene and the organic molecule, whilst the other (bottom) in vacuum conditions. Prior the optimization, the bottom surface has been passivated with hydrogen to prevent charge migration and saturate the dangling bonds of the cleaved surface.

The solvent has been included implicitly by means of the soft-sphere continuum solvation model^5,6^. In such approach the interface between the quantum-mechanical solute and surrounding environment is described by a fully continuum and differentiable permittivity ɛ(*r*), function of the atomic coordinates. The implicit solvent has been preferred to its explicit treatment in order to keep the size of the whole system affordable at DFT level, without neglecting the solid-liquid interaction. Mesitylene detains a dielectric constant of 2.265^7^. Despite its low value and apolar character which should not greatly affect vacuum geometries, a proper treatment needs its inclusion, especially for energetics evaluations. The solvation parameters for mesitylene provides a mean absolute error (MAE) of 0.71 kcal/mol over a set of experimental solvation free energies of small organic molecules (ɛ_0_ = 2.265, UFF radii $r_{i}^{vdW}$ with Bondi's radius for Nitrogen^8^, surface tension γ = 28.80 dyn/cm^9^, multiplying prefactor for radii *f* = 1.20, non-electrostatic energy terms with β = 0 GPa and α+γ = -12.0 dyn/cm). The isolate organic molecule diethyl-1-propyl phosphonate, DPP, (28 atoms) has been optimized in free boundary conditions both in vacuum and in mesitylene. Both ground states are similar, meaning that the inclusion of the solvent does not affect bond distances and angles of the plunged molecule. The computed solvation energy in mesitylene is -7.54 kcal/mol.

The binding energies for the various configurations discussed in the main text are reported in Table S1.

|  | 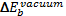 | 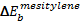 |
| --- | --- | --- |
| unbroken molecule | 0.063 | 0.197 |
| broken molecule (case I) | -3.22 | -3.04 |
| broken molecule (case II) | -0.52 | -0.31 |
| DFTB+MH global minimum | -3.23 | -3.00 |

**Supporting Table 1. |** **Binding energy values**. Binding energies [eV] in vacuum and mesitylene of the several molecule-surface configurations.

To explore the configurational space, we applied the structure prediction minima hopping (MH) method coupled to density functional tight binding (DFTB+)^10^. DFTB+ guarantees a faster computation of energies and forces with respect to *ab-initio* DFT, maintaining a reasonable accuracy. MH provides the exploration of the full configurational space, finding minimum energy structures by means of molecular dynamics trajectories^11^ and without restrictions on molecular bond breaking. For the local minimization within MH the stabilized quasi-Newton minimizer was used^12,13^. To extend the exploration of the phase space, the minima hopping search has been initialized from ten very dissimilar configurations (broken and not-broken molecule). All runs have been performed in vacuum. Results and output structures confirm that configurations with bond breaking are lower in energy with respect to the unbroken molecule. The surface tends to dissociate the organic molecule and the presence of the solvent globally alleviates molecule-surface bindings. The ten MH runs fell in ten distinct minimum. In order to get more accurate structures, to include the effect of solvent and allow energy comparisons, all DFTB+MH minima have been post-processed at *ab-initio* DFT level. The energetic order is not preserved passing from DFTB+ to DFT, except the lowest which is the more favorable in both approaches. This suggest that in principle all minima hopping minimum should be processed at DFT level and not only the global minimum. Such post-processing could help to find metastable configurations involved in the molecular doping dynamics.

**SUPPORTING REFERENCES**

1. Genovese, L., et al. Daubechies wavelets as a basis set for density functional pseudopotential calculations. *J. Chem. Phys.* **129**, 014109 (2008).

2. see BIGDFT website http://bigdft.org/Wiki/index.php?title=BigDFT_website.

3. Goedecker, S., Teter, M., and Hutter, J., Separable dual-space Gaussian pseudopotentials. *J. Phys. Rev. B* **54**, 1703-1710 (1996).

4. Willand, A. et al. Norm-conserving pseudopotentials with chemical accuracy compared to all electron calculations. *J. Chem. Phys.* **138**, 104109 (2013).

5. Fisicaro, G., Genovese, L., Andreussi, O., Marzari, N., and Goedecker, S. A generalized Poisson and Poisson-Boltzmann solver for electrostatic environments. *J. Chem. Phys.* **144**, 014103 (2016) .

6. Fisicaro, G., et al. Soft-Sphere Continuum Solvation in Electronic-Structure Calculations. *J. Chem. Theory Comput.* DOI: 10.1021/acs.jctc.7b00375 (2017).

7. Haynes, W. M. in *CRC Handbook of Chemistry and Physics* (CPC Press, Boca Raton, 2012).

8. Rappe, A. K., et al. Application of a universal force-field to organic-molecules. *J. Am. Chem. Soc.* **114**, 10035-10046 (1992).

9. Earhart, H. W., and Komin, A. P., “Polymethylbenzenes,” in Kirk-Othmer *Encyclopedia of Chemical Technology* (John Wiley & Sons, Inc., 2000).

10. Aradi, B., Hourahine, B., and Frauenheim, T. DFTB+, a sparse matrix-based implementation of the DFTB method. *J. Phys. Chem. A* **111**, 5678-5684 (2007).
11. Goedecker, S., “Minima hopping: An efficient search method for the global minimum of the potential energy surface of complex molecular systems”, *J. Chem. Phys.* **120,** 9911-9917 (2004).
12. B. Schaefer, S. A. Ghasemi, S. Roy, and S. Goedecker, Stabilized quasi-Newton optimization of noisy potential energy surfaces. *J. Chem. Phys.* **142**, 034112 (2015).
13. B. Schaefer and S. Goedecker, Computationally efficient characterization of potential energy surfaces based on fingerprint distances. *J. Chem. Phys.* **145**, 034101 (2016).
